# Longitudinal Relationship between the Introduction of Medicinal Cannabis and Polypharmacy: An Australian Real-World Evidence Study

**DOI:** 10.1155/2022/8535207

**Published:** 2022-11-07

**Authors:** Maja Kalaba, Graham M. L. Eglit, Matthew T. Feldner, Patrizia D. Washer, Tracie Ernenwein, Alistair W. Vickery, Mark A. Ware

**Affiliations:** ^1^Canopy Growth Corporation, 1 Hershey Drive, Smiths Falls, ON K7A 3K8, Canada; ^2^Emyria Limited, D2 661 Newcastle Street, Leederville WA 6007, PO Box 1442, West Leederville, WA 6901, Australia

## Abstract

**Background:**

Recent studies recommend medicinal cannabis (MC) as a potential treatment for chronic pain (CP) when conventional therapies are not successful; however, data from Australia is limited. This real-world evidence study explored how the introduction of MC related to concomitant medication use over time. Long-term safety also was examined.

**Methods:**

Data were collected by the Emerald Clinics (a network of seven clinics located across Australia) as part of routine practice from Jan 2020 toJan 2021. Medications were classified by group: antidepressants, benzodiazepines, nonsteroidal anti-inflammatory drugs (NSAIDs), opioids, and total number of medications. Adverse events (AEs) were collected at each visit and subsequently coded using the Medical Dictionary for Regulatory Activities version 23 into the system organ class (SOC) and preferred term (PT). A total of 535 patients were analyzed.

**Results:**

The most common daily oral dose was 10 mg for delta-9-tetrahydrocannabinol (THC) and 15 mg for cannabidiol (CBD). With the introduction of MC, patients' total number of medications consumed decreased over the course of one year; significant reductions in NSAIDs, benzodiazepines, and antidepressants were observed (*p* < .001). However, the number of prescribed opioid medications did not differ from baseline to the end of one year (*p* = .49). Only 6% of patients discontinued MC treatment during the study. A total of 600 AEs were reported in 310 patients during the reporting period and 97% of them were classified as nonserious. *Discussion*. Though observational in nature, these findings suggest MC is generally well-tolerated, consistent with the previous literature, and may reduce concomitant use of some medications. Due to study limitations, concomitant medication reductions cannot be causally attributed to MC. Nevertheless, these data underscore early signals that warrant further exploration in randomized trials.

## 1. Introduction

Australia legalized medicinal cannabis (MC) in 2016 [1]. There have been approvals for a wide range of conditions, including chronic pain (CP). In fact, tetrahydrocannabinol (THC)-containing cannabis for the treatment of CP is the most commonly prescribed MC product [2, 3]. CP causes significant psychological and physical burdens for patients and is difficult for clinicians to treat effectively [4]. The long-term safety and efficacy of opioids for the treatment of CP is controversial [5, 6]. Although combined drug therapy may improve pain management, adverse effects are common [7, 8]. Some preliminary evidence suggests that MC may improve pain-related outcomes [9, 10] and thus may be a viable substitution for opioids and polypharmacy for managing CP [9, 11–15]. For example, various surveys and observational studies, mainly in the United States, have shown that CP patients often reduce their use of traditional pain medications (e.g., opioids and benzodiazepines) upon initiation of MC. However, it is largely unknown whether CP patients in Australia may also substitute MC for traditional pain medications. Such knowledge may be important for general practitioners (GPs) in Australia, who often describe being poorly informed about potential uses of MC. [2, 16].

To explore the role of MC for CP and other indications, we designed a prospective real-world evidence study in which we monitored MC users (i.e., patients receiving a MC prescription under a physician's care). Our primary objective was to examine how the introduction of MC under medical supervision is related to concomitant medication use over time. Additionally, as an exploratory objective, we examined patient characteristics, dosing regimens, and long-term safety as a function of the cannabinoid profile.

## 2. Methods

### 2.1. Design & Setting

This is a prospective real-world evidence study of patients prescribed MC and followed up at the Emerald Clinic, a network of seven clinics located across Australia. Patients presenting to the Emerald Clinics are treated by specialists and GPs who have interests in chronic disease management and the appropriate use of unregistered medicines. At the initial visit, a consultation was completed to understand the patient's medical history and treatment goals. A series of standardized “pre-treatment” questionnaires was completed, and based on responses, the physician determined whether the patient was suitable for MC. Eligibility criteria included the following: (1) the patient is not pregnant or breastfeeding; (2) the patient has no severe or unstable mental health or cardiac conditions; (3) the patient has no suicidal thoughts or ideation; (4) the patient has exhausted other treatment options for clinical indication; and (5) the patient has a negative THC urine test at the baseline. If eligible, the physician completed a prescription and provided titration guidelines that included an initial dose and daily slow titration until an “effective” dose was achieved. The effective dose was decided on a case-by-case basis by the physician and patient and defined as a dose at which side effects were minimal and symptoms (e.g., pain relief) were adequately alleviated based on the treatment goals for a given patient. Patients and physicians were allowed to change the product or dose as clinically indicated during the study. In Australia, there are no standard guidelines for physicians to follow when prescribing MC. At the Emerald clinics, the following are taken into consideration when identifying which MC product and format to prescribe: (1) clinical indication (and the associated available literature to support the prescription), (2) age, (3) concomitant medications, (4) comorbidities, (5) driving restrictions, (6) previous experience with MC (prescribed or recreational), and (7) cost. MC products were dispensed by independent pharmacies, and on average, MC was dispensed within 24 hours of the baseline visit. In-person or telemedicine follow-up visits occurred on approximately a bimonthly basis to monitor the patient's health, adverse events, and adjust MC dosage and concomitant medications (as needed).

All registered patients signed an informed consent form and agreed to the use of their de-identified data for research purposes. Ethical approval by the human research committee was not required, as all assessments were conducted as part of routine clinical care in line with the Special Access Scheme requirements [1].

### 2.2. Sample

A total of 620 patients were enrolled between January 1^st^ 2020 and January 31^st^ 2021. To enable a prepost assessment of concomitant medication change subsequent to MC prescription, patients were required to have complete concomitant medication data, a baseline visit prior to beginning the use of MC, and at least one follow-up visit during which they were prescribed MC. Twenty-one patients were excluded for missing concomitant medication data, 4 patients for absence of MC-naïve baseline data, and 60 patients for lack of follow-up visit data. The final sample consisted of 535 patients.

### 2.3. Data Source

All data were collected through Emyria Digital Health, a Therapeutic Goods Administration (TGA)-registered data management platform. The following data were collected: demographics, primary diagnosis, MC prescription (product name, format, daily mgs of THC, and cannabidiol (CBD)), concomitant medications, and adverse events (AEs). We further classified products into three product profile categories (THC-dominant, CBD-dominant, and balanced). We included patients who were prescribed MC from one licensed producer (Spectrum Therapeutics) to standardize the exposure to products with known cannabinoid ratios. The list of Spectrum Therapeutics product offerings during the data collection period is shown in Table 1.

### 2.4. Outcomes

The following outcomes, assessed at baseline and bimonthly visits, were measured to address our primary and exploratory objectives. MC prescription was characterized in terms of (1) content of tetrahydrocannabinol (THC) and cannabidiol (CBD), (2) formulation (oral oil or softgel), and (3) average daily dose (mg of THC and CBD). Medications were classified by class, and the following categories were assessed: antidepressants, benzodiazepines, nonsteroidal anti-inflammatory drugs (NSAIDs), opioids, and total number of medications.  Table 2lists the medications within these categories. AEs were collected at each visit and subsequently coded using the Medical Dictionary for Regulatory Activities version 23 into the system organ class (SOC) and preferred term (PT) [17]. Seriousness criteria were defined by the International Conference of Harmonization. Irrespective of causality assessment, all AEs reported were included in the analysis, where an individual was consuming multiple products, and the AE reported was counted for each product.

### 2.5. Analyses to Address Primary Objective

Models for one-year change in the number of total medications and number of medications in specific classes (opioid, NSAIDs, etc.) were fit using generalized linear mixed-effect models. Truncated Conway–Maxwell–Poisson probability distributions were used owing to observed underdispersion and zero truncation/inflation on all outcomes. A fixed intercept was specified for the zero-inflated part of the model, and for the count component of the model, both nonlinear and linear change models were specified to determine the optimal functional form for the relation between time, days, and number of medications. Natural cubic splines with knots placed at the .25, .5, and .75 quantiles of the time variable (corresponding to days 21, 79, and 168 postbaseline) were used for nonlinear functional form estimation. To determine final model selection, nonlinear and linear change models were compared using likelihood ratio tests. To facilitate interpretation, linear contrast testing change in the number of medications at the baseline to the end of one year and corresponding percent change effect sizes with accompanying 95% Wald confidence intervals were calculated. All models included random slopes for time nested within the subject and implemented a natural logarithm link function. Maximum likelihood estimation with a Laplace approximation was used for model fitting. Alpha was set at .05, two-tailed.

R version 4.03 [18] was used for medication models. The glmmTMB package [19] was used for generalized linear mixed-effect models, the splines package [17] for spline modeling, and the emmeans package [20] for marginal mean estimation.

### 2.6. Analyses to Address Exploratory Objectives

Descriptive statistics were generated to describe patient characteristics, products prescribed, and AEs experienced. Summary statistics included the mean and standard deviation (SD) for continuous variables and counts and percentages for categorical variables.

## 3. Results

Table 3 presents descriptive statistics of the sample. Of the 535 patients who completed a baseline visit, 483 (90%), 357 (67%), 264 (49%), and 181 (33%) completed 2-, 4-, 6-, and 8-month follow-ups, respectively. Patient enrollment was ongoing throughout the year and was reflected in smaller samples at each follow-up. Only 36 patients (6%) discontinued MC during the study. Reasons for discontinuation were ineffective treatment (*n* = 18. 50%), other (*n* = 13. 36%), AEs (*n* = 6. 16%), and physician's decision (*n* = 1. 3%).

### 3.1. Baseline Products Prescribed

MC patients were predominantly prescribed MC oil (*n* = 476; 89%). Groupings of product profiles for oils were as follows: 66% (*n* = 351) balanced 1 : 1 (THC : CBD), 20% (*n* = 109) THC-dominant, 5% (*n* = 25) CBD-dominant, and 9% (*n* = 50) multiple products with different profiles. The daily dose ranged from 0.25 to 181 mg of THC and 0.15 to 400 mg of CBD. The most frequent daily dose post-titration was approximately 10 mg of THC and 15 mg of CBD (equivalent to 1 ml of oil per day). The remaining 11% of patients were prescribed either a THC-dominant softgel (5%) or a balanced softgel (6%). The daily dose ranged from 0.25 to10 mg of THC and 1 to 100 mg of CBD (equivalent to 1–4 softgels a day). Please refer to Table 1 for the list of Spectrum Therapeutics productsavailable during the data collection period.

### 3.2. Total Number of Concomitant Medications

**Figure 1:**
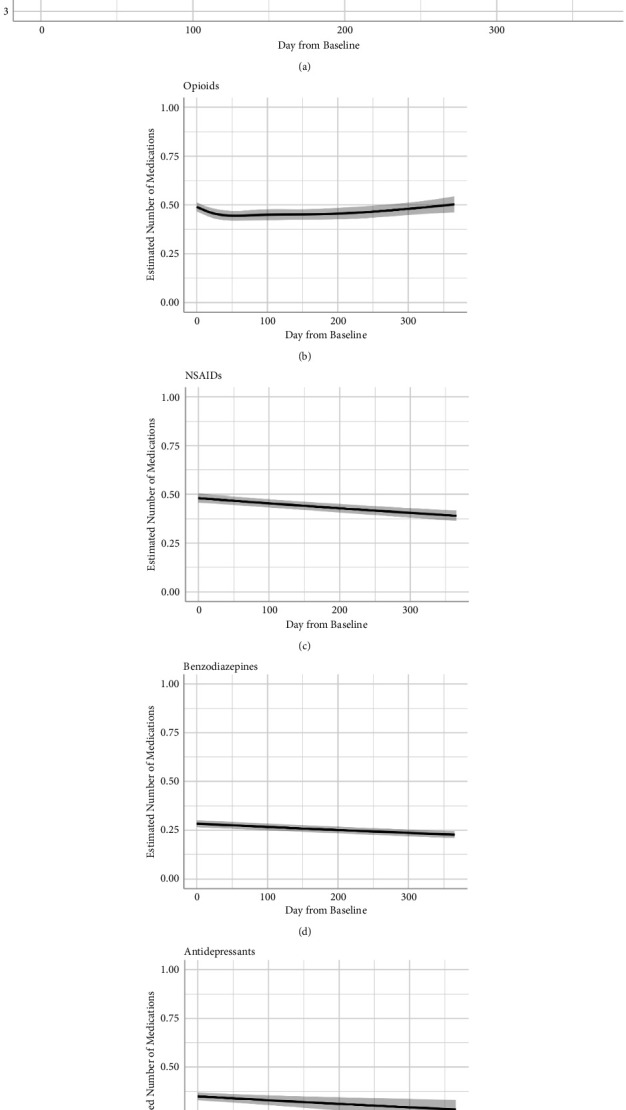
Estimated trajectories for one-year change in the number of total medications and number of medications in specific classes.

The nonlinear cubic spline model fit better than a simpler linear change model (*χ*^2^ = 50.14, *p* < .001).  Figure 1(a) presents the model estimates from the nonlinear model. Relative to the baseline, there was a 9.8% (95% CI = 7.04, 12.65) reduction in the mean number of total medications at six months and a 19.6% (95% CI = 14.0, 24.8) reduction at the end of one year (*t* = −6.38, *p* < .001).

### 3.3. Specific Medication Classes

For opioid medications, the nonlinear model fit better than a linear change model (*χ*^2^ = 23.52, *p* < .001). There was a 7.5% (95% CI = 2.48, 12.27) reduction in opioid medication at six months (*t* = −2.89, *p* = .004), but by the end of the year, patients used 2.6% (95% CI = −4.6, 10.4) more opioid medications, which was not a statistically significant change from baseline (*t* = 0.70, *p* = .49).

For NSAID medications, the nonlinear model did not fit better than a linear specification (*χ*^2^ = 3.09, *p* = .38), so the linear model was retained. As shown in Figure 1(c), there was a decrease in the number of NSAID medications over the course of the year. Relative to the baseline, patients used estimated 9.89% (95% CI = 7.04, 12.65) fewer NSAID medications at six months and 18.8% fewer (95% CI = 13.58, 23.7) at the end of one year (*t* = −6.55, *p* < .001).

Change in the number of benzodiazepine medications was best estimated using a linear change model (*χ*^2^ = 6.20, *p* = .30). There was a decline in benzodiazepine use over the course of the year, with patients using estimated 8.5% (95% CI = 6.35, 10.74) fewer benzodiazepines at six months and 16.4% (95% CI = 12.3, 20.3) fewer benzodiazepine medications (*t* = −7.32, *p* < .001) (see Figure 1(d)).

Change in the number of antidepressant medications was also best estimated using a linear change model (*χ*^2^ = 3.46, *p* = .33). Relative to the baseline, there was a decrease in antidepressant medications, with patients using estimated 10.16% (95% CI = 2.54, 17.18) fewer antidepressants at six months and 19.3% (95% CI = 5.02, 31.41) fewer antidepressants at the end of one year (*t* = −2.58, *p* = .01; Figure 1(e)).

### 3.4. Safety

**Figure 2:**
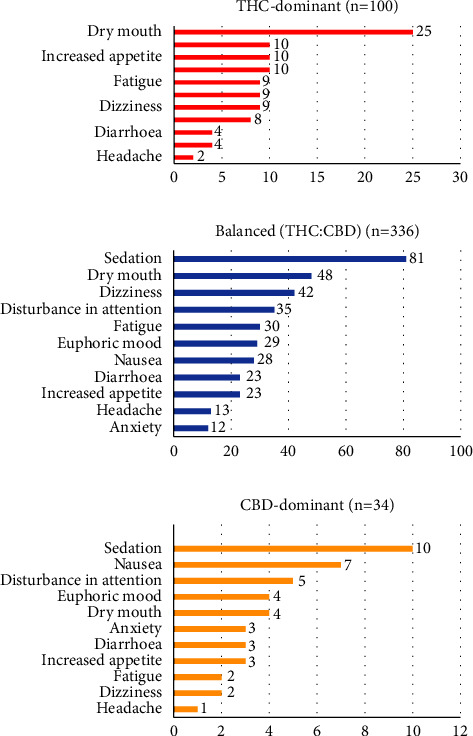
Distribution of most frequent nonserious AEs (PT level) per product profile.

A total of 600 AEs were reported across 310 patients during the reporting period. Ninety-seven percent of AEs were nonserious. Causality assessment was only available for 35 cases (5%).  Figure 2 displays the distribution of the most frequent nonserious AEs based on the cannabinoid profile. Hallucinations (*n* = 17; 2%) were the most prevalent serious AE, followed by seizures (*n* = 2; <1%). Note that seizure cases occurred in patients who had a prior history of seizures and were taking anticonvulsants throughout the observation period. Both patients were using MC for chronic pain, and neither indicated the use of MC to help manage seizures. One was prescribed spectrum red and the other was prescribed spectrum blue.

## 4. Discussion

Two major findings emerged from this real-world study. First, among MC users, the number of medications that patients used decreased over time. Consistent with prior studies, initiation of MC resulted in reductions in use of NSAIDs, benzodiazepines, and antidepressants over the course of the year [9, 11, 13]. However, unlike some prior observational studies [9, 14, 21], MC initiation initially reduced but then reverted to the baseline number of opioid medications across the year follow-up period. Second, MC products were well tolerated.

It appears that when patients initiate MC use, there is a correlated reduction in use of several other medications. This suggests that there may be a role of MC in the treatment of chronic pain and comorbidities. Future research should try to better elucidate why MC may substitute for pain medications and explore if there are potential ancillary benefits of MC for daily management of CP (i.e., improved quality of life). Interestingly, although there was a brief initial reduction of opioid medication, by the end of the year, no long-term reduction in opioid use was observed in contrast to prior work [9, 14]. Studies of opioid reduction as the primary focus are needed to understand this discrepancy and determine if there are individual or population-specific factors that dictate whether MC substitutes for opioids. This discrepancy may be that this study measured the number of medications taken and not the total dose of medication. It should also be noted that there were fewer cases at later time points due to the rolling recruitment nature of data collection, so estimation of the number of opioid medications at later time points was less precise than at earlier times. Nevertheless, these findings generally align with emerging evidence, suggesting that the use of prescription drugs may be decreasing in countries where cannabis is available [11–13, 22–24].

Most AEs experienced were nonserious, suggesting that MC is well tolerated; this is consistent with a systematic review that found 96.6% of MC AEs were not serious [25]. Cannabis is not without its harms; for instance, with repeated use, some individuals may develop cannabis use disorder [26]. Moreover, though CBD and THC are relatively safe [27], they are not risk free, and the potential for drug-drug interactions must be considered [28]. However, unlike THC and CBD, opioids can be lethal at high doses, which necessitates prescribing low opioid doses for pain [29–31].

Real-world evidence reflects clinical experience across a large and diverse distribution of patients, providing insights into real-world treatment patterns. However, this study design is not without limitations. First, the lack of a control group precludes ruling out regression to the mean, placebo effects, and secular trends, among other biases, contributing to change in concomitant medication use over time. As such, concomitant medication reductions cannot be causally attributed to MC. Second, due to continuous enrollment, there were considerably less data available at later time points. Natural cubic splines were used to avoid overfitting to time points with sparse data and to mitigate the influence of these sparse data regions on estimates at earlier time points. Nonetheless, there was greater uncertainty in medication use estimates at later time points. Third, this study did not evaluate the effectiveness of cannabinoids vs other medications, and no conclusion can be drawn regarding sustained pain relief with cannabinoids.

## 5. Conclusion

These prospective real-world data reveal insights into MC practices, including use, safety, and relation to polypharmacy over a one-year period. MC taken at various doses appears to be well tolerated, and significant reductions in polypharmacy over time were observed. While recognizing that real-world studies have limitations, these data underscore early signals that warrant further exploration.

## Figures and Tables

**Table 1:** Spectrum therapeutics product avalibility in Australia.

Spectrum products	Product profile (THC:CBD ratio)	Percentage of THC & CBD per Gram of weight dried flower (%)	Milligram of THC & CBD per millilitre of oil (mg/mL)	Milligram of THC & CBD per softgel (mg)
Red	THC-dominant (2 : 1)	16 THC: <1 CBD	26.3 THC: <1 CBD	2.5 or 10 THC: <1 CBD
Blue	Balanced (1 : 1)	NA	10 THC: 12–15 CBD	2.5 THC: 3.75 CBD
Yellow	CBD-dominant (1 : 2)	NA	<1 THC: 20 CBD	<1 THC: 20 CBD

**Table 2:** Classification of medications (generic + brand names).

Classification of medications (generic + brand names)			
Antidepressants	Benzodiazepines	NSAIDs	Opioids
Agomelatine (Valdoxan)	Alprazolam (Xanax, Kalma)	Advil	Buprenorphine (buvidal, norspan, Suboxone)

Amitriptyline	Clonazepam (Paxam)	Arthrexin	Codapane forte

Bupropion (Zyban)	Diazepam (Antenex, Valium, Valpam)	Arthro-Aid	Codeine

Clomipramine	Flunitrazepam	Aspirin	Fentanyl (Abstral, Durogesic) hydromorphone (Dilaudid, Jurnista)

Cymbalta (Duloxetine)	Lorazepam (Ativan)	Celecoxib (Celebrex)	Ketamine

Deptran (Doxepin)	Midazolam	Diclofenac (Voltaren)	Mersyndol

Desvenlafaxine (Pristiq)	Nitrazepam (Alodorm, Mogadon)	Etoricoxib (Acorxia)	Methadone (Physeptone)

Dosulepin (Prothiaden)	Oxazepam (Alepam, Serepax)	Fenac	Morphine (MS Contin)

Effexor (Venlafaxine)	Temazepam tramadol (Normison)	Ibuprofen (Nurofen)	Ondansetron (Zofran)

Fluoxetine (Prozac)		Indomethacin	Oxycodone (Endone, OxyContin, Oxynorm, Proladone)

Mirtazapine nortriptyline (Pamelor)		Ketoprofen	Panadeine

Sertraline (Zoloft)		Ketorolac injection	Tapentadol (Palexia)

Trazodone		Maxigesic (Ibuprofen, Paracetamol)	Targin
		Melobic	Temgesic
		Meloxicam (Mobic)	Tramadol
		Movalis	
		Naproxen	
		Nuromol	
		Osteomol paracetamol	
		Paracetamol (Acetaminophen, Panadol)	
		Piroxicam (Feldene)	
		Proxen SR	

**Table 3:** Baseline characteristics (*n* = 535).

Baseline characteristics	n (%)
Female	279 (52)
Age (mean (SD))	57.2 (17.8)
Age range (years)	9–94
Number of medications (mean (SD))^1^	7.06 (4.38)
State of residence	
Western Australia	356 (67)
New South Wales	155 (29)
Victoria	24 (4)
Employment	
Employed status	196 (36)
Full-time employed	117
Part-time employed	79
Unemployed status	339 (64)
Retired	140
Unable to work due to pain	103
Unable to work due to a condition other than pain	57
Not working by choice (e.g., student and homemaker)	28
Other	11
Primary indication	
Chronic noncancer pain	Epilepsy
Cancer pain	45 (8)
Other indications	18 (3)
Insomnia	14 (3)
Parkinson's disease	11 (2)
Post-traumatic stress disorder	8
Neurological spasticity	8
Chemotherapy induced nausea and vomiting	8
Migraine (headache)	7
Alzheimer's or dementia	4
Inflammatory bowel disease	1
Epilepsy	1

Notes. ^1^Most common baseline medication classes used were opioids (60.6%), nonsteroidal anti-inflammatory drugs (54.4%), antidepressants (36.8%), benzodiazepines (34.6%), and proton pump inhibitors (32.7%).

## Data Availability

The dataset used and/or analyzed during the current study is available from the corresponding author on reasonable request.
